# 1,3-Bis(2-chloro­phen­yl)thio­urea: a monoclinic polymorph

**DOI:** 10.1107/S1600536811041894

**Published:** 2011-10-12

**Authors:** Chien Ing Yeo, Edward R. T. Tiekink

**Affiliations:** a Department of Chemistry, University of Malaya, 50603 Kuala Lumpur, Malaysia

## Abstract

The title compound, C_13_H_10_Cl_2_N_2_S, represents a monoclinic polymorph of the previously reported ortho­rhom­bic form [Ramnathan *et al.* (1996 ▶). *Acta Cryst*. C**52**, 134–136]. The mol­ecule is twisted with the dihedral angle between the benzene rings being 55.37 (7)°. The N—H atoms are *syn* to each other, which contrasts their *anti* disposition in the ortho­rhom­bic form. In the crystal, mol­ecules assemble into zigzag chains along the *c* axis *via* N—H⋯S hydrogen bonds. Chains are connected into layers *via* C—H⋯Cl inter­actions, and these stack along the *a* axis.

## Related literature

For background to the structural chemistry of thio­carbamides, see: Ho *et al.* (2005 ▶). For a related diaryl­thio­urea structure, see: Kuan & Tiekink (2007 ▶). For the structure of the ortho­rhom­bic polymorph, see: Ramnathan *et al.* (1996 ▶).
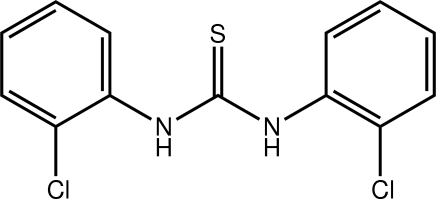


## Experimental

### 

#### Crystal data

C_13_H_10_Cl_2_N_2_S
*M* *_r_* = 297.19
Monoclinic, 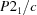

*a* = 11.9999 (3) Å
*b* = 14.6811 (3) Å
*c* = 8.0806 (2) Å
β = 109.509 (1)°
*V* = 1341.84 (5) Å^3^
*Z* = 4
Mo *K*α radiation
μ = 0.62 mm^−1^
*T* = 100 K
0.20 × 0.06 × 0.02 mm

#### Data collection

Bruker SMART APEX diffractometer
Absorption correction: multi-scan (*SADABS*; Sheldrick, 1996 ▶) *T*_min_ = 0.644, *T*_max_ = 0.746
12491 measured reflections
3090 independent reflections
2291 reflections with *I* > 2σ(*I*)
*R*_int_ = 0.052

#### Refinement

*R*[*F* ^2^ > 2σ(*F* ^2^)] = 0.038
*wR*(*F* ^2^) = 0.090
*S* = 1.02
3090 reflections
169 parameters
2 restraints
H atoms treated by a mixture of independent and constrained refinement
Δρ_max_ = 0.35 e Å^−3^
Δρ_min_ = −0.43 e Å^−3^

### 

Data collection: *APEX2* (Bruker, 2009 ▶); cell refinement: *SAINT* (Bruker, 2009 ▶); data reduction: *SAINT*; program(s) used to solve structure: *SHELXS97* (Sheldrick, 2008 ▶); program(s) used to refine structure: *SHELXL97* (Sheldrick, 2008 ▶); molecular graphics: *ORTEP-3* (Farrugia, 1997 ▶), *DIAMOND* (Brandenburg, 2006 ▶) and *Qmol* (Gans & Shalloway, 2001 ▶); software used to prepare material for publication: *publCIF* (Westrip, 2010 ▶).

## Supplementary Material

Crystal structure: contains datablock(s) global, I. DOI: 10.1107/S1600536811041894/vm2125sup1.cif

Structure factors: contains datablock(s) I. DOI: 10.1107/S1600536811041894/vm2125Isup2.hkl

Supplementary material file. DOI: 10.1107/S1600536811041894/vm2125Isup3.cml

Additional supplementary materials:  crystallographic information; 3D view; checkCIF report

## Figures and Tables

**Table 1:** Hydrogen-bond geometry (Å, °)

*D*—H⋯*A*	*D*—H	H⋯*A*	*D*⋯*A*	*D*—H⋯*A*
N1—H1n⋯S1^i^	0.87 (2)	2.62 (2)	3.4449 (18)	159 (2)
N2—H2n⋯S1^i^	0.87 (2)	2.49 (2)	3.3389 (18)	166 (2)
C13—H13⋯Cl2^ii^	0.95	2.79	3.660 (2)	152

Symmetry codes: (i) 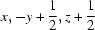; (ii) 
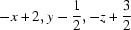
.

**Fig. 1.**
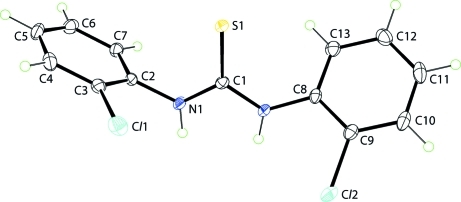
The molecular structure of (I) showing the atom-labelling scheme and displacement ellipsoids at the 50% probability level.

**Fig. 2.**
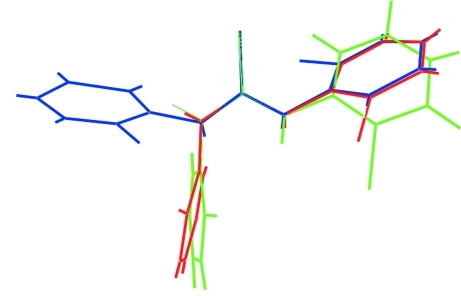
Overlay diagram of (I) (shown in blue) with the two independent molecules found in the orthorhombic polymorph (see text), shown in red and green. The central thiourea fragments have been fitted.

**Fig. 3.**
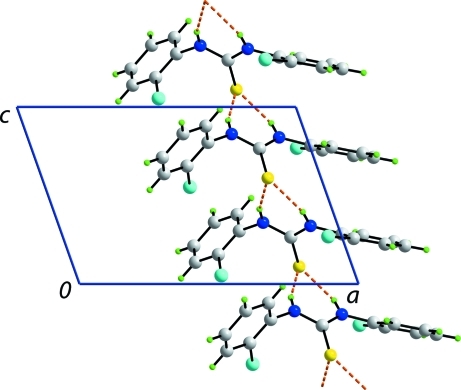
Supramolecular zigzag chain in (I) mediated by N—H···S hydrogen bonds, shown as orange dashed lines.

**Fig. 4.**
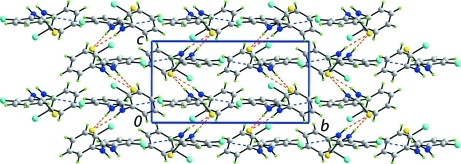
A view of the supramolecular two-dimensional array in the bc plane for (I). The N—H···S and C—H···Cl contacts are shown as orange and blue dashed lines, respectively.

**Fig. 5.**
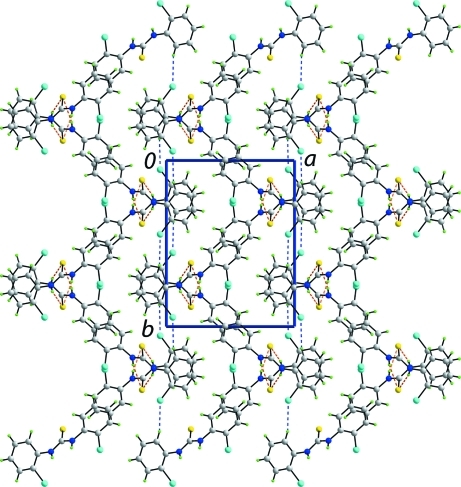
Unit-cell contents for (I) shown in projection down the c axis, showing the stacking of supramolecular layers along the a axis. The N—H···S and C—H···Cl contacts are shown as orange and blue dashed lines, respectively.

## supplementary crystallographic information

### Comment

In connection with the synthesis and structural studies of thiocarbamides (Ho *et al.*, 2005), diarylthioureas are sometimes isolated as the undesired hydrolysis by-product (Kuan & Tiekink, 2007). The title compound, (I), was isolated in crystalline form during the attempted synthesis of (C_6_H_4_Cl-*o*)N(H)C(═S)O-*i*-Pr. The structure of (I) is reported herein. A previously reported orthorhombic form of (I) exists in the literature (Ramnathan *et al.*, 1996).

The molecular structure of (I), Fig. 1, is twisted about the central N—C bonds. With reference to the central plane through the CN_2_S chromophore [r.m.s. deviation = 0.0029 Å], the C2-benzene ring is almost perpendicular [dihedral angle = 85.20 (6)°] and the C8-ring is twisted [dihedral angle = 49.32 (6)°]; the dihedral angle between the benzene rings is 55.37 (7)°. The amide-H atoms are *syn* to each other. The *syn* conformation observed for the thiourea chromophore in (I) is quite distinct to that found in the orthorhombic polymorph (Ramnathan *et al.*, 1996).

In the orthorhombic form of (I), the amide-H atoms are *anti* to each other. This key difference between the molecular structures in the two polymorphs is highlighted in the overlay diagram shown in Fig. 2. The *anti* orientation allows for the formation of eight-membered {···HNCS}_2_ synthons in the crystal packing in the orthorhombic polymorph. By contrast, the crystal structure of (I) features supramolecular zigzag chains along the *c* axis mediated by N—H···S hydrogen bonds (Table 1 and Fig. 3); the S1 atom is bifurcated. Chains assemble into layers by C—H···Cl interactions (Fig. 4) and the layers thus formed stack along the *a* axis (Fig. 5).

### Experimental

2-Chlorophenyl isothiocyanate (2 ml) was added drop-wise to a stirred solution of NaOH (1 mol equiv.) in i-PrOH (25 ml) and stirred for 3 h. Excess HCl (50% *M*/v) was then added and the solution was stirred for a further 2 h. The product was then extracted with CHCl_3_ and left for evaporation at room temperature yielding colourless crystals after 3 days; *M*.pt: 394–396 K. IR (cm^-1^): ν(N—H) 3348; ν(C—N) 1498; ν(C═ S) 1201.

### Refinement

Carbon-bound H-atoms were placed in calculated positions (C—H 0.95 Å) and were included in the refinement in the riding model approximation, with *U*_iso_(H) set to 1.2*U*_eq_(C). The N-bound H-atoms were located in a difference Fourier map but were refined with a distance restraint of N—H = 0.88±0.01 Å, and with *U*_iso_(H) = 1.2*U*_eq_(N).

### Crystal data

**Table:**

C_13_H_10_Cl_2_N_2_S	*F*(000) = 608
*M**_r_* = 297.19	*D*_x_ = 1.471 Mg m^−^^3^
Monoclinic, *P*2_1_/*c*	Mo *K*α radiation, λ = 0.71073 Å
Hall symbol: -P 2ybc	Cell parameters from 2105 reflections
*a* = 11.9999 (3) Å	θ = 2.3–25.4°
*b* = 14.6811 (3) Å	µ = 0.62 mm^−^^1^
*c* = 8.0806 (2) Å	*T* = 100 K
β = 109.509 (1)°	Prism, colourless
*V* = 1341.84 (5) Å^3^	0.20 × 0.06 × 0.02 mm
*Z* = 4	

### Data collection

**Table:**

Bruker SMART APEX diffractometer	3090 independent reflections
Radiation source: fine-focus sealed tube	2291 reflections with *I* > 2σ(*I*)
graphite	*R*_int_ = 0.052
ω scans	θ_max_ = 27.5°, θ_min_ = 2.3°
Absorption correction: multi-scan (*SADABS*; Sheldrick, 1996)	*h* = −15→15
*T*_min_ = 0.644, *T*_max_ = 0.746	*k* = −19→19
12491 measured reflections	*l* = −10→10

### Refinement

**Table:**

Refinement on *F*^2^	Primary atom site location: structure-invariant direct methods
Least-squares matrix: full	Secondary atom site location: difference Fourier map
*R*[*F*^2^ > 2σ(*F*^2^)] = 0.038	Hydrogen site location: inferred from neighbouring sites
*wR*(*F*^2^) = 0.090	H atoms treated by a mixture of independent and constrained refinement
*S* = 1.02	*w* = 1/[σ^2^(*F*_o_^2^) + (0.0375*P*)^2^ + 0.4482*P*] where *P* = (*F*_o_^2^ + 2*F*_c_^2^)/3
3090 reflections	(Δ/σ)_max_ < 0.001
169 parameters	Δρ_max_ = 0.35 e Å^−^^3^
2 restraints	Δρ_min_ = −0.43 e Å^−^^3^

### Special details

**Table:**

Geometry. All s.u.'s (except the s.u. in the dihedral angle between two l.s. planes) are estimated using the full covariance matrix. The cell s.u.'s are taken into account individually in the estimation of s.u.'s in distances, angles and torsion angles; correlations between s.u.'s in cell parameters are only used when they are defined by crystal symmetry. An approximate (isotropic) treatment of cell s.u.'s is used for estimating s.u.'s involving l.s. planes.
Refinement. Refinement of *F*^2^ against ALL reflections. The weighted *R*-factor *wR* and goodness of fit *S* are based on *F*^2^, conventional *R*-factors *R* are based on *F*, with *F* set to zero for negative *F*^2^. The threshold expression of *F*^2^ > 2σ(*F*^2^) is used only for calculating *R*-factors(gt) *etc*. and is not relevant to the choice of reflections for refinement. *R*-factors based on *F*^2^ are statistically about twice as large as those based on *F*, and *R*- factors based on ALL data will be even larger.

### Fractional atomic coordinates and isotropic or equivalent isotropic displacement parameters (Å^2^)

**Table:**

	*x*	*y*	*z*	*U*_iso_*/*U*_eq_	
Cl2	0.95110 (5)	0.44450 (4)	0.76829 (8)	0.03177 (16)	
Cl1	0.51862 (5)	0.24151 (4)	0.54764 (9)	0.03412 (17)	
S1	0.80893 (5)	0.11833 (4)	0.59792 (7)	0.01789 (13)	
N1	0.73545 (16)	0.18714 (12)	0.8464 (2)	0.0200 (4)	
H1N	0.740 (2)	0.2301 (12)	0.923 (2)	0.024*	
N2	0.90127 (15)	0.25343 (12)	0.8279 (2)	0.0189 (4)	
H2N	0.891 (2)	0.2893 (13)	0.907 (2)	0.023*	
C1	0.81669 (17)	0.18925 (14)	0.7647 (3)	0.0171 (4)	
C2	0.63988 (18)	0.12309 (15)	0.7997 (3)	0.0192 (4)	
C3	0.53473 (19)	0.14084 (15)	0.6660 (3)	0.0223 (5)	
C4	0.4428 (2)	0.07777 (17)	0.6227 (3)	0.0280 (5)	
H4	0.3708	0.0904	0.5311	0.034*	
C5	0.4575 (2)	−0.00316 (17)	0.7142 (3)	0.0290 (6)	
H5	0.3955	−0.0468	0.6845	0.035*	
C6	0.5615 (2)	−0.02114 (16)	0.8484 (3)	0.0274 (5)	
H6	0.5708	−0.0769	0.9112	0.033*	
C7	0.6528 (2)	0.04214 (15)	0.8918 (3)	0.0223 (5)	
H7	0.7242	0.0298	0.9849	0.027*	
C8	1.00456 (18)	0.26531 (15)	0.7822 (3)	0.0190 (5)	
C9	1.03988 (19)	0.35296 (15)	0.7568 (3)	0.0227 (5)	
C10	1.1449 (2)	0.36828 (17)	0.7252 (3)	0.0292 (6)	
H10	1.1687	0.4286	0.7104	0.035*	
C11	1.2144 (2)	0.2954 (2)	0.7154 (3)	0.0340 (6)	
H11	1.2861	0.3054	0.6927	0.041*	
C12	1.1804 (2)	0.20762 (18)	0.7386 (3)	0.0308 (6)	
H12	1.2284	0.1575	0.7303	0.037*	
C13	1.07637 (19)	0.19243 (16)	0.7739 (3)	0.0248 (5)	
H13	1.0543	0.1321	0.7925	0.030*	

### Atomic displacement parameters (Å^2^)

**Table:**

	*U*^11^	*U*^22^	*U*^33^	*U*^12^	*U*^13^	*U*^23^
Cl2	0.0352 (3)	0.0165 (3)	0.0443 (4)	−0.0053 (2)	0.0142 (3)	0.0001 (3)
Cl1	0.0332 (3)	0.0314 (3)	0.0386 (4)	0.0093 (3)	0.0131 (3)	0.0113 (3)
S1	0.0192 (3)	0.0152 (3)	0.0199 (3)	−0.0022 (2)	0.0072 (2)	−0.0024 (2)
N1	0.0229 (9)	0.0184 (9)	0.0211 (10)	−0.0073 (8)	0.0105 (8)	−0.0070 (8)
N2	0.0195 (9)	0.0151 (9)	0.0231 (10)	−0.0049 (7)	0.0083 (8)	−0.0045 (7)
C1	0.0179 (10)	0.0135 (10)	0.0179 (11)	0.0006 (8)	0.0034 (8)	0.0024 (8)
C2	0.0181 (10)	0.0202 (11)	0.0229 (11)	−0.0039 (9)	0.0115 (9)	−0.0062 (9)
C3	0.0243 (12)	0.0221 (12)	0.0242 (12)	−0.0004 (9)	0.0130 (9)	−0.0013 (9)
C4	0.0197 (11)	0.0412 (15)	0.0239 (12)	−0.0041 (10)	0.0083 (9)	−0.0069 (11)
C5	0.0287 (13)	0.0330 (14)	0.0307 (13)	−0.0163 (11)	0.0170 (11)	−0.0129 (11)
C6	0.0358 (13)	0.0212 (12)	0.0312 (13)	−0.0065 (10)	0.0193 (11)	−0.0015 (10)
C7	0.0228 (11)	0.0225 (11)	0.0232 (12)	−0.0032 (9)	0.0099 (9)	−0.0019 (9)
C8	0.0161 (10)	0.0221 (11)	0.0176 (11)	−0.0049 (9)	0.0041 (8)	−0.0021 (9)
C9	0.0234 (11)	0.0225 (11)	0.0206 (11)	−0.0055 (9)	0.0051 (9)	−0.0026 (9)
C10	0.0287 (13)	0.0324 (14)	0.0274 (13)	−0.0157 (11)	0.0104 (10)	−0.0035 (11)
C11	0.0210 (12)	0.0528 (17)	0.0298 (14)	−0.0131 (12)	0.0108 (10)	−0.0079 (12)
C12	0.0210 (12)	0.0378 (14)	0.0325 (14)	0.0013 (11)	0.0075 (10)	−0.0076 (11)
C13	0.0202 (11)	0.0252 (12)	0.0261 (12)	−0.0016 (10)	0.0037 (9)	−0.0023 (10)

### Geometric parameters (Å, °)

**Table:**

Cl2—C9	1.737 (2)	C5—H5	0.9500
Cl1—C3	1.736 (2)	C6—C7	1.389 (3)
S1—C1	1.681 (2)	C6—H6	0.9500
N1—C1	1.348 (3)	C7—H7	0.9500
N1—C2	1.433 (3)	C8—C9	1.391 (3)
N1—H1N	0.871 (10)	C8—C13	1.389 (3)
N2—C1	1.354 (3)	C9—C10	1.385 (3)
N2—C8	1.417 (3)	C10—C11	1.375 (4)
N2—H2N	0.872 (9)	C10—H10	0.9500
C2—C7	1.383 (3)	C11—C12	1.383 (4)
C2—C3	1.384 (3)	C11—H11	0.9500
C3—C4	1.392 (3)	C12—C13	1.388 (3)
C4—C5	1.379 (3)	C12—H12	0.9500
C4—H4	0.9500	C13—H13	0.9500
C5—C6	1.379 (3)		
			
C1—N1—C2	122.16 (18)	C7—C6—H6	120.0
C1—N1—H1N	116.2 (16)	C2—C7—C6	120.1 (2)
C2—N1—H1N	121.4 (16)	C2—C7—H7	120.0
C1—N2—C8	126.73 (18)	C6—C7—H7	120.0
C1—N2—H2N	115.1 (15)	C9—C8—C13	118.7 (2)
C8—N2—H2N	118.1 (15)	C9—C8—N2	119.22 (19)
N1—C1—N2	113.90 (18)	C13—C8—N2	121.9 (2)
N1—C1—S1	121.53 (16)	C10—C9—C8	121.2 (2)
N2—C1—S1	124.56 (16)	C10—C9—Cl2	119.76 (18)
C7—C2—C3	119.4 (2)	C8—C9—Cl2	119.03 (17)
C7—C2—N1	119.08 (19)	C11—C10—C9	119.4 (2)
C3—C2—N1	121.5 (2)	C11—C10—H10	120.3
C2—C3—C4	120.7 (2)	C9—C10—H10	120.3
C2—C3—Cl1	119.68 (17)	C10—C11—C12	120.4 (2)
C4—C3—Cl1	119.65 (18)	C10—C11—H11	119.8
C5—C4—C3	119.3 (2)	C12—C11—H11	119.8
C5—C4—H4	120.3	C11—C12—C13	120.2 (2)
C3—C4—H4	120.3	C11—C12—H12	119.9
C4—C5—C6	120.4 (2)	C13—C12—H12	119.9
C4—C5—H5	119.8	C12—C13—C8	120.1 (2)
C6—C5—H5	119.8	C12—C13—H13	120.0
C5—C6—C7	120.1 (2)	C8—C13—H13	120.0
C5—C6—H6	120.0		

### Hydrogen-bond geometry (Å, °)

**Table:**

*D*—H···*A*	*D*—H	H···*A*	*D*···*A*	*D*—H···*A*
N1—H1n···S1^i^	0.873 (17)	2.618 (17)	3.4449 (18)	158.6 (19)
N2—H2n···S1^i^	0.868 (18)	2.49 (2)	3.3389 (18)	166 (2)
C13—H13···Cl2^ii^	0.95	2.79	3.660 (2)	152

Symmetry codes: (i) *x*, −*y*+1/2, *z*+1/2; (ii) −*x*+2, *y*−1/2, −*z*+3/2.
